# Complete Chloroplast Genome Sequence and Phylogenetic Analysis of *Paeonia ostii*

**DOI:** 10.3390/molecules23020246

**Published:** 2018-01-26

**Authors:** Shuai Guo, Lili Guo, Wei Zhao, Jiang Xu, Yuying Li, Xiaoyan Zhang, Xiaofeng Shen, Mingli Wu, Xiaogai Hou

**Affiliations:** 1 College of Agricultural (College of Tree Peony), Henan University of Science and Technology, Luoyang 471023, Henan, China; shuai93guo@163.com (S.G.); guolili0928@163.com (L.G.); zhwibcas@163.com (W.Z.); 160314310517@stu.haust.edu.cn (Y.L.); 2 Institute of Chinese Materia Medical, China Academy of Chinese Medical Sciences, Beijing 100700, China; jxu@icmm.ac.cn (J.X.); xyzhangkind@126.com (X.Z); 18384253631@163.com (X.S.); justuswu@163.com (M.W.); 3 College of Life Science, Huaibei Normal University, Huaibei 235000, Anhui, China; 4 College of Pharmacy, Hubei University of Chinese Medicine, Wuhan 430065, Hubei China

**Keywords:** *Paeonia ostii*, chloroplast genome, phylogeny

## Abstract

*Paeonia ostii*, a common oil-tree peony, is important ornamentally and medicinally. However, there are few studies on the chloroplast genome of *Paeonia ostii*. We sequenced and analyzed the complete chloroplast genome of *P. ostii*. The size of the *P. ostii* chloroplast genome is 152,153 bp, including a large single-copy region (85,373 bp), a small single-copy region (17,054 bp), and a pair of inverted repeats regions (24,863 bp). The *P. ostii* chloroplast genome encodes 111 genes, including 77 protein-coding genes, four ribosomal RNA genes, and 30 transfer RNA genes. The genome contains forward repeats (22), palindromic repeats (28), and tandem repeats (24). The presence of rich simple-sequence repeat loci in the genome provides opportunities for future population genetics work for breeding new varieties. A phylogenetic analysis showed that *P. ostii* is more closely related to *Paeonia delavayi* and *Paeonia* *ludlowii* than to *Paeonia* *obovata* and *Paeonia* *veitchii*. The results of this study provide an assembly of the whole chloroplast genome of *P. ostii*, which may be useful for future breeding and further biological discoveries. It will provide a theoretical basis for the improvement of peony yield and the determination of phylogenetic status.

## 1. Introduction

The tree peony (*Paeonia suffruticosa* Andrews), a woody shrub, belongs to the section Moutan in the genus *Paeonia*, family Paeoniaceae [1]. Tree peony has been grown for approximately 1400 years and is an important ornamental and medicinal plant aboriginal to China [2,3,4]. In recent years, with advances in research on tree peony, a number of studies have been conducted on the fatty acids in tree peony seed oil [5,6,7,8,9,10,11].

Oil peony is among the new woody oil crops and can be used to produce seeds for processing into edible peony oil. Through years of research, experimentation and exploration, experts have found two oil-producing varieties in the existing peony population, namely *Paeonia rockii* and *Paeonia ostii.* These two varieties of seed-oil peony significantly outnumber the other varieties, and the value of their oil is well known. Because peony oil contains large concentrations of alpha-linolenic acid and unsaturated fatty acids, it has a certain preventive effect on cardiovascular disease [12,13,14]. In 2011, the Ministry of Health of the People’s Republic of China issued a notice to consider peony seed oil as a new food resource. In 2014, the China Food and Drug Administration listed peony seed oil on the list of available cosmetics, indicating that peony seed oil could be formally entered into the edible oil and cosmetics markets. However, due to limited planting area, the total output of peony seed oil is not high, resulting in peony seed oil processing and production costs being relatively high. Therefore, it would be desirable to improve grain yield by increasing plant photosynthetic capacity [15]. 

Chloroplasts, the descendants of ancient bacterial endosymbionts, are significant, active organelles in plant cells responsible for photosynthesis and myriad other aspects of metabolism [16]. The DNA of chloroplasts (cpDNA) is independent of the nuclear genome, showing semi-autonomous genetic characteristics. Chloroplast genome size differences are large; for example, microtubule plant chloroplast DNA is generally 120–160 kb, and the size of the phytoplankton chloroplast genome is about 120 kb [17]. The structure of the chloroplast genome is a typical four-segment, double-stranded, cyclic molecular structure, with one large single-copy (LSC) region, one small single-copy (SSC) region and two inverse repeats (IRs) regions with substantially the same length [18]. Plant chloroplast genomes generally have 110 to 130 genes that are highly conserved in their composition and arrangement within the genome, including photosynthetic genes, chloroplast transcriptional expression-related genes, and some other protein-coding genes [19]. As the center of photosynthesis, the study of the chloroplast genome is of great significance to reveal the mechanisms and metabolic regulation of plant photosynthesis. Using the differences of light systems of plants, we can improve the efficiency of light absorption and transformation, and further improve plant yield [20]. At the same time, the characteristics of maternally inherited genes and highly conserved genes of the chloroplast genome provide favorable conditions for studying the phylogenetic status of plants.

Studies of the genome of the peony chloroplast currently remain limited to phylogenetic analysis using some chloroplast genes [4,21,22,23,24,25], and no peony chloroplast whole-genome information has been reported. Here we describe the whole chloroplast genome sequence of *P. ostii*, together with the characterization of long repeats and simple sequence repeats (SSRs). We compared and analyzed the chloroplast genome of *P. ostii* and the chloroplast genome of other members of the genus *Paeonia*. It is expected that the results will provide a theoretical basis for the improvement of peony yield and the determination of phylogenetic status.

## 2. Results and Discussion 

### 2.1. Features of P. ostii cpDNA

More than 50 million paired-end reads were produced by the Illumina Hiseq X Ten sequencing platform for *P. ostii*. After reference-guided assembly, the complete chloroplast genome sequence of *P. ostii* was obtained and submitted to the NCBI database with the GenBank accession number MG585274. The chloroplast genome size is 152,153 bp. The structure of the *P. ostii* chloroplast genome is similar to those from the other *Paeonia* species [26], including an LSC region (85,373 bp; covering 56.1%), an SSC region (17,054 bp; covering 11.2%), and a pair of inverted repeats (IRA/IRB, 24,863 bp; covering 16.3%) (Table 1). The DNA G+C content of the LSC, SSC and IR regions, and the whole genome, is 36.7, 32.7, 43.1 and 38.4 mol %, respectively, which is also similar to the chloroplast genomes of other *Paeonia* species. The DNA G+C content is a very important indicator to judge species affinity, and *P. ostii* has a similar cpDNA G+C content to other *Paeonia* species [27,28,29]. The DNA G+C content of the IR regions is higher than that of other regions (LSC, SSC); this phenomenon is very common in other plants [18,27]. The relatively high DNA G+C content of the IR regions was mostly attributable to the rRNA genes and tRNA genes [18,30].

**Figure 1:**
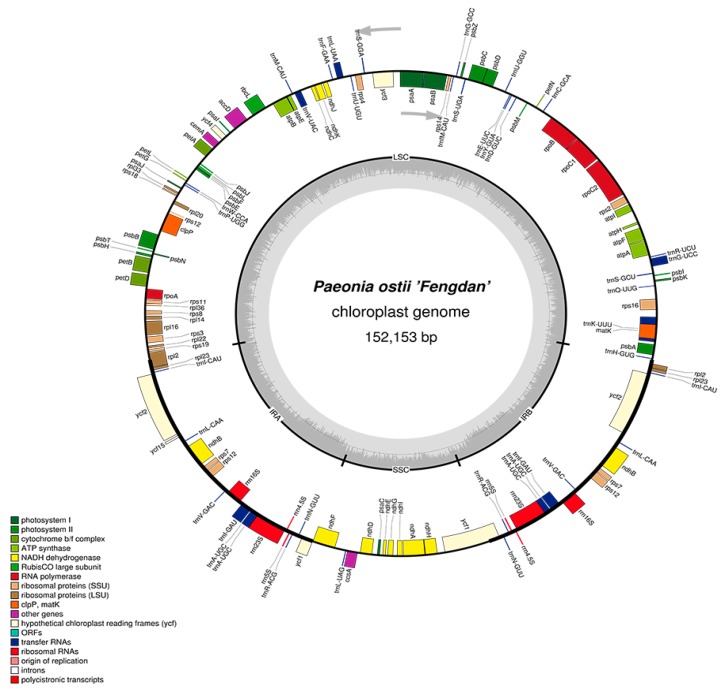
Gene map of the *P. ostii* chloroplast genome. Genes drawn inside the circle are transcribed clockwise, and those outsides are transcribed counterclockwise. Genes belonging to different functional groups are color-coded. The darker gray in the inner circle corresponds to DNA G+C content, while the lighter gray corresponds to A+T content. The gray arrowheads indicate the direction of the genes.

In the *P. ostii* chloroplast genome, 111 functional genes were predicted, including four rRNA genes, 30 tRNA genes, and 77 protein-coding genes (Table 2). In addition, 18 genes—seven tRNA, all four rRNA and seven protein-coding genes—are duplicated in the IR regions (Figure 1). The LSC region includes 58 protein-coding and 22 tRNA genes, while the SSC region includes one tRNA gene and 11 protein-coding genes.

The sequences of the tRNA and protein-coding genes were analyzed, and the frequency of codon usage was deduced for the *P. ostii* chloroplast genome and summarized. A total of 33,967 codons represent the coding capacity of 77 protein-coding and tRNA genes in *P. ostii* (Table 3). Among these codons, 3728 (10.98%) encode for leucine and 626 (1.83%) for tryptophan, which are the most and least prevalent amino acids in the *P. ostii* chloroplast genome, respectively. A- and U-ending codons were common [18,31].

There are, altogether, 18 intron-containing genes, including 12 protein-coding genes and six tRNA genes (Table 4). Fifteen genes (nine protein-coding and six tRNA genes) contain one intron, and two genes (*ycf3* and *clpP*) contain two introns (Table 4). The intron of the *trnK-UUU* gene contains the *matK* gene, and the size of the intron is 2452 bp. The *rps12* gene is a trans-spliced gene, with the 5’ end located in the LSC region and the duplicated 3’ ends in the IR regions. Previous studies have reported that *ycf3* is required for the stable accumulation of the photosystem I complex [32,33]. We therefore speculate that the intron gain in *ycf3* of *P. ostii* may be useful for further studies of the mechanism of photosynthesis evolution.

Advances in phylogenetic research have shown that chloroplast genome evolution includes structural changes and nucleotide substitutions [34,35]. A few examples of these changes, including intron or gene losses, have been found in chloroplast genomes [36,37,38,39,40,41]. The introns are significant in the regulation of gene expression [42]. They can improve gene expression level, on the special position and in the specific time [43,44]. Studies of intron regulation mechanisms have appeared in other species [45,46]. However, no studies analyzing associations between intron loss and gene expression, using transcriptome data from *P. ostii*, have been published. More experimental work is required to study the introns in *P. ostii*, in order to establish a theoretical foundation for improving the production of oil.

### 2.2. Long-Repeat and SSR Analysis

**Figure 2:**
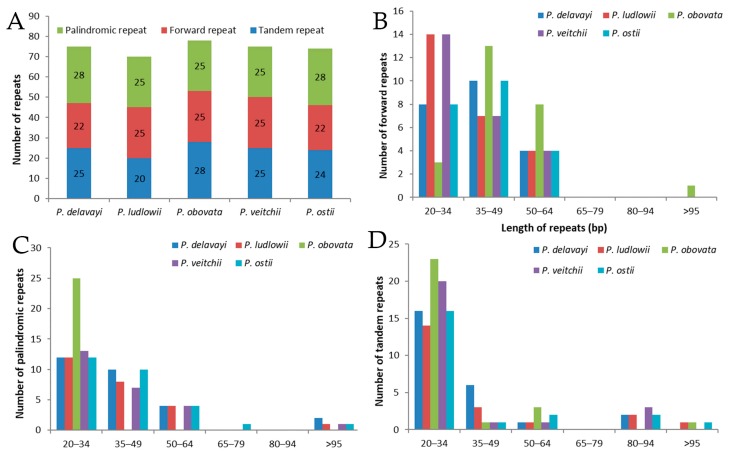
Analysis of repeated sequences in five *Paeonia* chloroplast genomes. (**A**) Totals of three repeat types; (**B**) frequency of forward repeats by length; (**C**) frequency of palindromic repeats by length; (**D**) frequency of tandem repeats by length.

A total of 74 repeats were detected in the *P. ostii* chloroplast genome, including 22 forward repeats, 28 palindromic repeats, and 24 tandem repeats (Figure 2). Among these, most of the forward repeats are 20–64 bp in length, and 19 tandem repeats and 26 palindromic repeats are of similar length (Figure 2B–D). Similarly, 75, 70, 78 and 75 repeat pairs were found in the previously reported *P. delavayi*, *P. ludlowii*, *P. obovata* and *P. veitchii* chloroplast genomes, respectively. This suggests that *P. ostii* is more similar to *P. delavayi* with respect to number of repeats.

**Figure 3:**
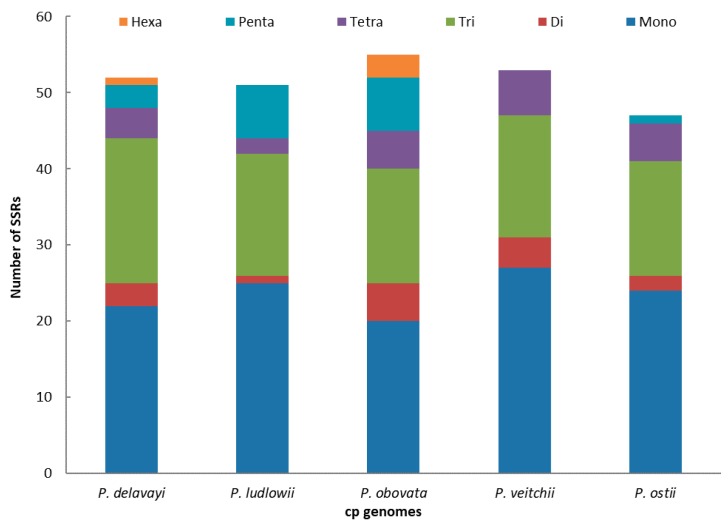
Analysis of simple sequence repeats (SSRs) in the five *Paeonia* chloroplast genomes.

SSRs of 10 bp or longer are inclined to undergo slipped-strand mispairing, which is identified as the main mutational mechanism of SSR polymorphisms [27]. SSRs in the chloroplast genome can be highly variable at the intraspecific level and are therefore often used as genetic markers in population genetics and evolutionary studies [47,48,49,50]. In this study, we found SSRs in the chloroplast genome of *P. ostii* together with those of four other *Paeonia* species (Figure 3). The numbers of SSRs are 52, 51, 55, 53 and 47, respectively, in *P. delavayi*, *P. ludlowii*, *P. obovata*, *P. veitchii* and *P. ostii.* The mononucleotide repeat content is the largest (*P. delavayi*, 42.30%*; P. ludlowii*, 49.02%*; P obovata*, 36.36%; *P. veitchii*, 50.94%; *P. ostii*, 51.06%) in all the above species. However, dinucleotides are the least frequent repeat type in the five species (*P. delavayi*, 1.92%; *P. ludlowii*, 0%*; P. obovata*, 5.54%; *P. veitchii*, 0%; *P. ostii*, 0%) (Figure 3). These results will afford chloroplast SSR markers that can be used to study genetic diversity and related species in *P. ostii*; this also provides an effective method to select germplasm for high-yield oil.

### 2.3. Comparative Chloroplast Genomic Analysis

**Figure 4:**
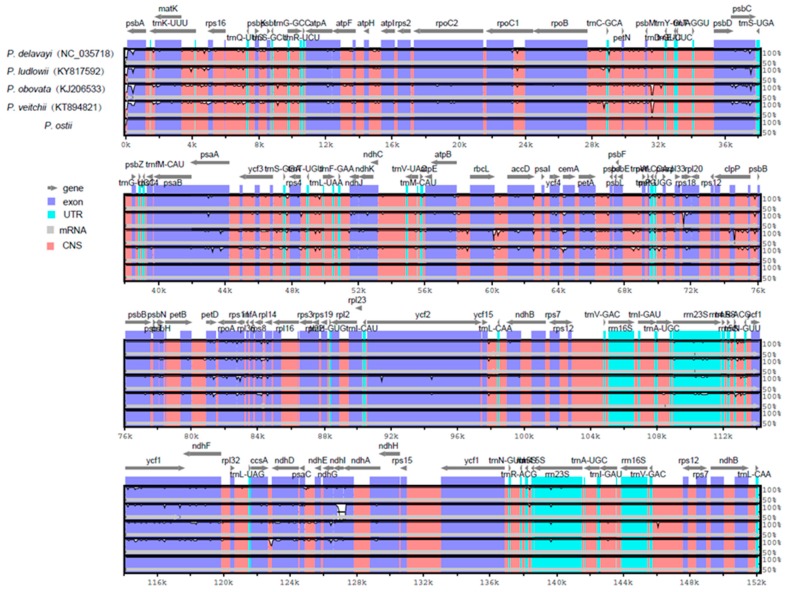
Comparison of five chloroplast genomes using mVISTA. Gray arrows and thick black lines above the alignment indicate gene orientation. Purple bars represent exons, blue bars represent untranslated regions (UTRs), pink bars represent noncoding sequences (CNS), gray bars represent mRNA, and white peaks represent differences of genomics. The y-axis represents the percentage identity (shown: 50–100%).

Comparative analysis of chloroplast genomes is an extremely important step in genomics [51,52]. Comparing the structural differences between *Paeonia* chloroplast genomes revealed that the chloroplast genome size of *P. ostii* is the smallest among the five completed *Paeonia* chloroplast genomes (Table 1). *P. ostii* has the smallest IR regions (24,863 bp) among these sequenced *Paeonia* chloroplast genomes. We surmised that the different length of the IR regions is the main reason for the change in sequence length. To explain the level of genome divergence, sequence identity among *Paeonia* chloroplast DNAs was calculated using the program mVISTA with *P. ostii* as a reference (Figure 4). The results of this comparison revealed that the IR (A/B) regions are less divergent than the LSC and SSC regions. Furthermore, the noncoding regions are more variable than the coding regions, and the highly divergent regions among the five chloroplast genomes occur in the intergenic spacers [53].

### 2.4. IR Contraction and Expansion in the P. ostii Chloroplast Genome

**Figure 5:**
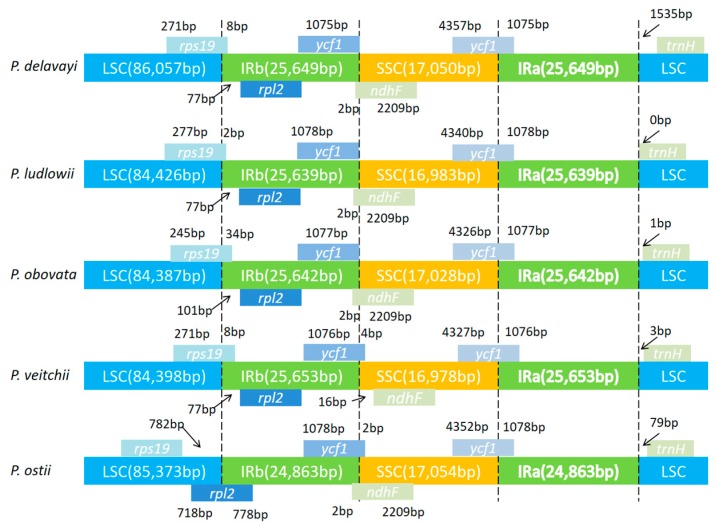
Comparison of border distance between adjacent genes and junctions of the LSC, SSC and two IR regions among the chloroplast genomes of five *Paeonia* species. Boxes above or below the main line indicate the adjacent border genes. The figure is not to scale with respect to sequence length, and only shows relative changes at or near the IR/SC borders.

Contractions and expansions of the IR regions at the borders are common evolutionary events and represent the main reasons for the size variation of chloroplast genomes; they play an important role in evolution [54,55,56]. A detailed comparison of four junctions, LSC/IRA (JLA), LSC/IRB (JLB), SSC/IRA (JSA) and SSC/IRB (JSB), between the two IRs (IRA and IRB) and the two single–copy regions (LSC and SSC), was completed among *P. delavayi*, *P. ludlowii*, *P. obovata*, *P. veitchii*, and *P. ostii* (Figure 5)*.* The SSC/IRA junction is located in the *ycf1* region in all *Paeonia* species chloroplast genomes and extends a different length (*P. delavayi*, 4357 bp; *P. ludlowii*, 4340 bp; *P. obovata*, 4326 bp; *P. veitchii*, 4327 bp; *P. ostii*, 4352 bp) into the SSC region in all genomes; the IRB region includes 1075, 1078, 1077, 1076 and 1078 bp of the *ycf1* gene, respectively. Recently, it was reported that *ycf1* is necessary for plant viability and encodes Tic214, an important component of the *Arabidopsis* TIC complex [57,58]. Similarly, the *trnH* gene is located in the LSC region, 1535, 0, 1, 3 and 79 bp away from the IRA/LSC border in the five *Paeonia* chloroplast genomes, respectively. The JLA junction in *P. ostii* is crossed by *rpl2*, which is different from the other four *Paeonia* species. The *ndhF* gene is across the JSB junction in *P. delavayi*, *P. ludlowii*, *P. obovata* and *P. ostii*, while it was found to be 16 bp away from the IRB/SSC border in *P. veitchii.*

Although the gene order in chloroplasts is usually conserved in terrestrial plants, it has been reported that many sequences are rearranged in chloroplast genomes from a wide variety of different plant species, including inversions in the LSC region, IR contraction or expansions with inversions, and re-inversion in the SSC region [59,60,61,62,63]. Sequence rearrangements that change chloroplast genome structure in connected species may also deliver genetic diversity information that can be used for molecular classification and evolution studies.

### 2.5. Phylogenetic Analysis

**Figure 6:**
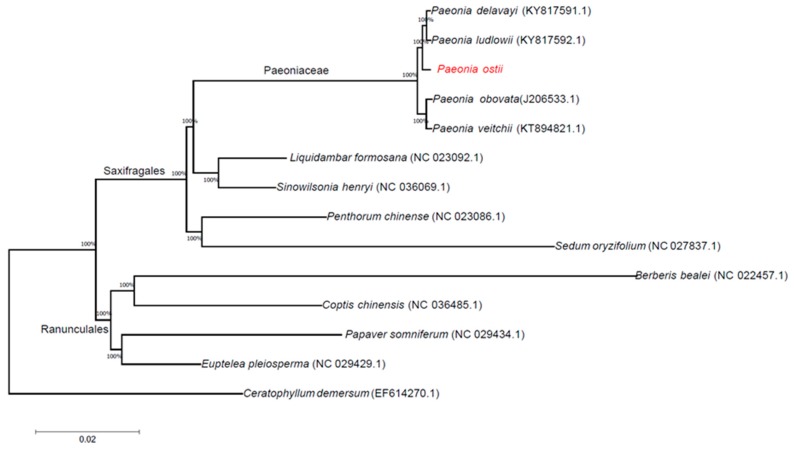
ML phylogenetic tree reconstruction including 14 species based on all chloroplast genomes. The position of *P. ostii* is indicated in red font. *Ceratophyllum demersum* was used as the outgroup.

Phylogenetic analyses were completed on an alignment of concatenated nucleotide sequences of all chloroplast genomes from 14 angiosperm species. We used the method of maximum likelihood (ML) to build a phylogenetic tree based on these chloroplast genome data, and *Ceratophyllum demersum* was used as the outgroup (Figure 6). The ML phylogenetic results powerfully supported the hypothesis that all *Paeonia* species form a subgroup. *P. delavayi* and *P. ludlowii* are sister species; *P. ostii* is closer to *P. delavayi* and *P. ludlowii* than to *P. obovata* and *P. veitchii*. According to the phylogenetic analysis of chloroplast genomes, the Paeoniaceae family belongs to the Saxifragales rather than the Ranunculales.

## 3. Materials and Methods 

### 3.1. DNA Sequencing, Chloroplast Genome Assembly, and Validation 

The oil-tree peony used was *P. ostii*, planted in the experimental field of Henan University of Science and Technology in Luoyang, China (N 34°44′, E 112°27′). Fresh *P. ostii* leaves were collected and wrapped with tin foil, frozen by liquid nitrogen, and stored in a −80 °C preservation reserve. A modified cetyltrimethylammonium bromide (CTAB) method was used to extract the whole genomic DNA of *P. ostii* [64]. The concentration of DNA was checked by using a ND-2000 spectrometer (Nanodrop Technologies, Wilmington, DE, USA). The library type was a 250 bp shotgun library according to the manufacturer’s instructions (Vazyme Biotech Co. Ltd., Nanjing, China). The library was sequenced by the Illumina Hiseq X Ten platform double terminal sequencing method. The amount of data from the sample was 7.5 G; the total number of raw reads was 50 million (SRA accession: SRP129874).

The raw data was filtered by Skewer-0.2.2 [65]. BLAST searches were used to extract chloroplast-like reads from clean reads in comparison with reference sequences (*P. ludlowii*) [66]. Finally, we used the chloroplast-like reads to assemble sequences by using SOAPdenovo-2.04 [67]. SSPACE-3.0 and GapCloser-1.12 were used to extend sequences and fill gaps [68,69]. PCR amplification and Sanger sequencing were used to check the four junction regions between the IR regions and LSC/SSC to confirm the assembly (Table S1).

### 3.2. Gene Annotation and Sequence Analyses

CpGAVAS was used to annotate the sequences; DOGMA (http://dogma.ccbb.utexas.edu/) and BLAST were used to check the results of annotation [70,71]. tRNAscanSEv1.21 (http://lowelab.ucsc.edu/tRNAscan-SE/), with default settings, was used to identify all tRNA genes [70]. OGDRAWv1.2 (http://ogdraw.mpimp-golm.mpg.de/) was used to illustrate the structural features of chloroplast genomes [72]. Relative synonymous codon usage (RSCU) values were determined by MEGA5.2 (Department of Biological Sciences, Tokyo, Japan) [73].

### 3.3. Genome Comparison

The program mVISTA (Shuffle-LAGAN mode) was used to compare the whole chloroplast genome of *P. ostii* with the whole chloroplast genomes of *P. delavayi*, *P. ludlowii*, *P. obovata* and *P. veitchii* (KY817591, KY817592, KJ206533, KT894821) with the annotation of *P. ostii* as the reference [74,75]. The SSRs and forward (inverted) repeats were detected by Tandem Repeats Finder (Department of Biomathematical Sciences, New York, NY, USA) and REPuter individually (https://tandem.bu.edu/trf/trf.html) [76,77]. Phobos version 3.3.12 [78] was used to detect (SSRs) within the cp genome, with the search parameters set at ≥10 repeat units for mononucleotides, ≥8 repeat units for dinucleotides, ≥4 repeat units for trinucleotides and tetranucleotides, and ≥3 repeat units for pentanucleotide and hexanucleotide SSRs.

### 3.4. Phylogenetic Analysis

We downloaded 13 whole chloroplast genome sequences from the NCBI Organelle Genome and Nucleotide Resources database and used all genomes to analyze the phylogenetics. The software clustalw2 (The Conway Institute of Biomolecular and Biomedical Research, Dublin, Ireland) was used to align the genome [79]. MEGA5.2 (Department of Biological Sciences, Tokyo, Japan) [73] was used to analyze and plot the phylogenetic tree with ML (maximum likelihood). Bootstrap analysis was executed with 1000 replicates and TBR branch swapping. We used 1000 replicates and TBR branch exchange to complete the bootstrap analysis. Furthermore, *Ceratophyllum demersum* was set as the outgroup.

## 4. Conclusions

In summary, we present the first complete chloroplast genome of *P. ostii*, an important plant used for ornamental and medicinal purposes and for its oil. The genome sequencing, assembly, annotation and comparative analysis revealed that the chloroplast genome of *P. ostii* has a quadruple structure, gene order, DNA G+C content, and codon usage features similar to those of other *Paeonia* species’ chloroplast genomes. Compared with the chloroplast genomes of four related *Paeonia* species, the chloroplast genome size of *P. ostii* is the smallest, while the genome structure and composition are similar. Phylogenetic relationships among six *Paeonia* species revealed that *P. ostii* is more closely related to *P. delavayi* and *P. ludlowii* than to *P. obovata* and *P. veitchii.* The results of this study provide an assembly of a whole chloroplast genome of *P. ostii*, which may be useful for future breeding and further biological discoveries. It will provide a theoretical basis for the improvement of peony yield and the determination of phylogenetic status.

## Figures and Tables

**Table 1:** Summary of complete chloroplast genomes for five *Paeonia* species.

Species	*P. delavayi*	*P. ludlowii*	*P. obovata*	*P. veitchii*	*P. ostii*
**LSC (large single-copy)**					
**Length (bp)**	86,057	84,426	84,387	84,398	85,373
**G+C (%)**	36.7	36.7	36.7	36.7	36.7
**Length (%)**	56.4	55.3	55.3	55.3	56.1
**SSC (small single-copy)**					
**Length (bp)**	17,050	16,983	17,028	16,978	17,054
**G+C (%)**	32.7	32.7	32.7	32.7	32.7
**Length (%)**	11.2	11.1	11.2	11.1	11.2
**IR (inverse repeats)**					
**Length (bp)**	25,649	25,639	26,642	25,653	24,863
**G+C (%)**	43.1	43.1	43.1	43.1	43.1
**Length (%)**	16.8	16.8	17.4	16.8	16.3
**Total**					
**Length (bp)**	152,698	152,687	152,698	152,682	152,153
**G+C (%)**	38.4	38.4	38.4	38.4	38.4

**Table 2:** List of genes in the *P. ostii* chloroplast genome.

Category	Group of Genes	Name of Genes
Self-replication	Large subunit of ribosomal proteins	*rpl2* *^,a^, *14*, *16* *, *20*, *22*, *23* ^a^, *33*, *36*
	Small subunit of ribosomal proteins	*rps2*, *3*, *4*, *7* ^a^, *8*, *11*, *12* *^,a^, *14*, *16* *, *18*, *19*
	DNA-dependent RNA polymerase	*rpoA*, *B*, *C1* *, *C2*
	rRNA genes	*rrn16S* ^a^, *rrn23S* ^a^, *rrn4.5S* ^a^, *rrn5S* ^a^
	tRNA genes	*trnA-UGC* *^,a^, *trnC-GCA*, *trnD-GUC*, *trnE-UUC*, *trnF-GAA*, *trnfM-CAU*, *trnG-UCC* *, *trnG-GCC*, *trnH-GUG*, *trnI-CAU*, *trnI-GAU* *^,a^, *trnK-UUU* *, *trnL-CAA*, *trnL-UAA* *, *trnL-UAG*, *trnM-CAU*, *trnN-GUU*, *trnP-UGG*, *trnQ-UUG*, *trnR-ACG*, *trnR-UCU*, *trnS-GCU*, *trnS-GGA*, *trnS-UGA*, *trnT-GGU*, *trnT-UGU*, *trnV-GAC*, *trnV-UAC* *, *trnW-CCA*, *trnY-GUA*
Photosynthesis	Photosystem I	*psaA*, *B*, *C*, *I*, *J*
	Photosystem II	*psbA*, *B*, *C*, *D*, *E*, *F*, *H*, *I*, *J*, *K*, *L*, *M*, *N*, *T*, *Z*,
	NADH oxidoreductase	*ndhA* *, *B* *^,a^, *C*, *D*, *E*, *F*, *G*, *H*, *I*, *J*, *K*
	Cytochrome b6/f complex	*petA*, *B* *, *D* *, *G*, *L*, *N*
	ATP synthase	*atpA*, *B*, *E*, *F* *, *H*, *I*
	Rubisco	*rbcL*
Other genes	Maturase	*matK*
	Protease	*clpP* *
	Envelope membrane protein	*cemA*
	Subunit acetyl-CoA-carboxylase	*accD*
	c-Type cytochrome synthesis gene	*ccsA*
	Conserved open reading frames	*ycf1*, 2 ^a^, 3 *, 4, 15

* Genes containing introns; ^a^ duplicated gene (genes present in the IR regions).

**Table 3:** Codon–anticodon recognition patterns and codon usage of the *P. ostii* chloroplast genome.

Amino Acid	Codon	No.	RSCU *	tRNA	Amino Acid	Codon	No.	RSCU *	tRNA
Phe	UUU	1226	1.16		Tyr	UAU	937	1.36	
Phe	UUC	885	0.84	*trnF-GAA*	Tyr	UAC	440	0.64	*trnY-GUA*
Leu	UUA	769	1.24	*trnL-UAA*	Stop	UAA	683	1.1	
Leu	UUG	831	1.34	*trnL-CAA*	Stop	UAG	604	0.97	
Leu	CUU	690	1.11		His	CAU	486	1.34	
Leu	CUC	483	0.78		His	CAC	240	0.66	*trnH-GUG*
Leu	CUA	563	0.91	*trnL-UAG*	Gln	CAA	703	1.27	*trnQ-UUG*
Leu	CUG	392	0.63		Gln	CAG	401	0.73	
Ile	AUU	1055	1.19		Asn	AAU	1033	1.33	
Ile	AUC	746	0.84	*trnI-GAU*	Asn	AAC	520	0.67	*trnN-GUU*
Ile	AUA	855	0.97	*trnI-UAU*	Lys	AAA	1334	1.24	*trnK-UUU*
Met	AUG	743	1	*trn(f)M-CAU*	Lys	AAG	817	0.76	
Val	GUU	564	1.27		Asp	GAU	705	1.46	
Val	GUC	293	0.66	*trnV-GAC*	Asp	GAC	264	0.54	*trnD-GUC*
Val	GUA	548	1.24	*trnV-UAC*	Glu	GAA	923	1.27	*trnE-UUC*
Val	GUG	366	0.83		Glu	GAG	526	0.73	
Ser	UCU	659	1.45		Cys	UGU	393	1.19	
Ser	UCC	458	1.01	*trnS-GGA*	Cys	UGC	270	0.81	*trnC-GCA*
Ser	UCA	599	1.32	*trnS-UGA*	Stop	UGA	580	0.93	
Ser	UCG	388	0.85		Trp	UGG	626	1	*trnW-CCA*
Pro	CCU	368	1.02		Arg	CGU	217	0.58	*trnR-ACG*
Pro	CCC	309	0.86	*trnP-GGG*	Arg	CGC	182	0.49	
Pro	CCA	427	1.18	*trnP-UGG*	Arg	CGA	400	1.07	
Pro	CCG	341	0.94		Arg	CGG	372	1	
Thr	ACU	382	1.15		Arg	AGA	330	0.73	*trnR-UCU*
Thr	ACC	290	0.87	*trnT-GGU*	Arg	AGG	291	0.64	
Thr	ACA	407	1.23	*trnT-UGU*	Ser	AGU	632	1.69	
Thr	ACG	248	0.75		Ser	AGC	439	1.17	*trnS-GCU*
Ala	GCU	294	1.19		Gly	GGU	394	0.9	
Ala	GCC	227	0.92		Gly	GGC	261	0.6	*trnG-GCC*
Ala	GCA	253	1.02	*trnA-UGC*	Gly	GGA	579	1.33	*trnG-UCC*
Ala	GCG	217	0.88		Gly	GGG	509	1.17	

RSCU *: relative synonymous codon usage.

**Table 4:** Length of exons and introns in genes with introns in the *P. ostii* chloroplast genome.

Gene	Location	Exon I (bp)	Intron I (bp)	Exon II (bp)	Intron II (bp)	Exon III (bp)
*trnK-UUU*	LSC	35	2452	38		
*trnG-UCC*	LSC	23	709	49		
*trnL-UAA*	LSC	37	522	51		
*trnV-UAC*	LSC	38	576	39		
*trnI-GAU*	IR	42	935	36		
*trnA-UGC*	IR	37	42	29		
*rps12* *	LSC	26	543	227		114
*rps16*	LSC	234	820	40		
*rpl16*	LSC	402	1008	10		
*rpl2*	IR	435	670	394		
*rpoC1*	LSC	1617	709	436		
*ndhA*	SSC	540	1013	544		
*ndhB*	IR	756	684	778		
*ycf3*	SSC	153	765	229	721	126
*petB*	LSC	6	754	658		
*atpF*	LSC	408	701	160		
*clpP*	LSC	228	659	292	673	67
*petD*	LSC	9	645	526		

* The *rps12* gene is a trans-spliced gene with the 5’ end located in the LSC region and the duplicated 3’ ends in the IR regions.

## Supplementary Materials

Supplementary materials are available online. Table S1: Primers used for assembly validation.
