# Supplementary material for: Complete Chloroplast Genome Sequence and Phylogenetic Analysis of Paeonia ostii
Source: Molecules. 2018 Jan 26;23(2):246. doi: 10.3390/molecules23020246 (PMC6017096; doi:10.3390/molecules23020246)
Supplement: Supplementary file 1 [file molecules-23-00246-s001.pdf]

Table S1. Primers used for assembly validation.

Primer	Sequence (5’>3’)	Amplicon Size (bp)
LSC_IRa	TAGGAATCCAAATCTGCCGAA<br>ACCAATTAGGTTTACGACGAA	475
IRa_SSC	TTTAGGCAGAATACCGTCAC<br>AATAATTGATGGCATTACGAA	523
SSC_IRb	AAATATTGTGACATTTCGGTT<br>ATTTAGGCAGAATACCGTCA	406
IRb_LSC	CCAATTAGGTTTACGACGAA<br>TATGATGCCCTTCTTATTGGA	614
